# Identification of Autophagy-Related Genes and Their Regulatory miRNAs Associated with Celiac Disease in Children

**DOI:** 10.3390/ijms18020391

**Published:** 2017-02-12

**Authors:** Sergio Comincini, Federico Manai, Cristina Meazza, Sara Pagani, Carolina Martinelli, Noemi Pasqua, Gloria Pelizzo, Marco Biggiogera, Mauro Bozzola

**Affiliations:** 1 Department of Biology and Biotechnology, University of Pavia, 27100 Pavia, Italy; federico.manai01@universitadipavia.it (F.M.); carolina.martinelli01@universitadipavia.it (C.M.); marco.biggiogera@unipv.it (M.B.); 2 Pediatrics and Adolescentology Unit, Department of Internal Medicine and Therapeutics, University of Pavia, Fondazione IRCCS San Matteo, 27100 Pavia, Italy; c.meazza@smatteo.pv.it (C.M.); s.pagani@smatteo.pv.it (S.P.); mauro.bozzola@unipv.it (M.B.); 3 Department of Clinical-Surgical, Diagnostic and Pediatric Sciences, University of Pavia, Fondazione IRCCS San Matteo, 27100 Pavia, Italy; noemi.pasqua@hotmail.it (N.P.); g.pelizzo@smatteo.pv.it (G.P.)

**Keywords:** microRNA, autophagy, celiac disease molecular markers

## Abstract

Celiac disease (CD) is a severe genetic autoimmune disorder, affecting about one in 100 people, where the ingestion of gluten leads to damage in the small intestine. Diagnosing CD is quite complex and requires blood tests and intestinal biopsy examinations. Controversy exists regarding making the diagnosis without biopsy, due to the large spectrum of manifesting symptoms; furthermore, small-intestinal gastroscopy examinations have a relatively complex management in the pediatric population. To identify novel molecular markers useful to increase the sensitivity and specificity in the diagnosis of pediatric CD patients, the expression levels of two key autophagy executor genes (*ATG7* and *BECN1*) and their regulatory validated miRNAs (miR-17 and miR-30a, respectively) were analyzed by relative quantitative real-time-PCR on a cohort of confirmed CD patients compared to age-related controls. Among the investigated targets, the non-parametric Mann–Whitney *U* test and ROC analysis indicated the highest significant association of *BECN1* with CD status in the blood, while in intestinal biopsies, all of the investigated sequences were positively associated with CD diagnosis. Nomogram-based analysis showed nearly opposite expression trends in blood compared to intestine tissue, while hierarchical clustering dendrograms enabled identifying CD and control subgroups based on specific genes and miRNA expression signatures. Next, using an established in vitro approach, through digested gliadin administration in Caco-2 cells, we also highlighted that the modulation of miR-17 endogenous levels using enriched exosomes increased the intracellular autophagosome content, thereby altering the autophagic status. Altogether, these results highlighted novel molecular markers that might be useful to increase the accuracy in CD diagnosis and in molecular-based stratification of the patients, further reinforcing the functional involvement of the regulation of the autophagy process within a digestive and autoimmune-related disorder as CD.

## 1. Introduction

Celiac disease (CD), also referred to as celiac sprue and gluten-sensitive enteropathy, is a chronic systemic autoimmune disease of the small intestine. This disease results from an immune response, in genetically-susceptible individuals, to ingested dietary gluten and its related peptides in the form of wheat, barley and rye cereals and other environmental factors [1]. Small intestinal biopsy histology has long been considered an essential step for CD diagnosis. However, gastrointestinal endoscopy is invasive, costly and associated with risks for the patients. Researchers have therefore explored the possibility of CD diagnosis without endoscopy or biopsy analyses. In particular, European Society for Pediatric Gastroenterology, Hepatology and Nutrition (ESPGHAN) guidelines and other contributions proposed that in children and adolescents with signs or symptoms suggestive of CD and high anti-transglutaminase antibodies with levels ten-times higher compared to normal ones, the probability of villous atrophy is high, thus allowing CD diagnosis without biopsies’ examination [2,3].

Liquid biopsies based on circulating cell-free tumor DNA (ctDNA) or circulating miRNAs (cmiRNAs) hold great clinical promise for investigations in cancer and autoimmune disorders [4]. Several studies have uncovered possible roles for miRNA regulation in autoimmune diseases, specifically rheumatoid arthritis and systemic lupus erythematosus [5]. In another subset of inflammatory bowel diseases, it was reported that miRNAs’ expression signatures differentiate Crohn’s disease from ulcerative colitis (UC) [6]. Among these, miR-29a is overexpressed in the blood of UC patients, and it is also a potential non-invasive biomarker for colorectal cancer [7]. Benderska and colleagues [8] demonstrated that miR-26b overexpression at the intestinal level could serve as a biomarker to distinguish between ulcerative colitis-associated carcinogenesis and sporadic cancer types, showing that miRNAs can be useful in patients’ stratification.

The regulation of gene expression in the intestinal epithelium is complex and controlled by different signaling pathways that regulate the balance between proliferation and differentiation, processes altered in pathologies, such as CD [9]. To date, only a few studies have reported the identification of miRNAs as potential epigenetic biomarkers in CD [10]. Among these, Buoli Comani and colleagues [11] demonstrated that miRNAs and their gene targets showed an altered expression in duodenal mucosa and in plasma of CD pediatric patients, and these alterations could be different from adult ones. Specifically, it was reported that high levels of miR-449a targeted and reduced both NOTCH1 and KLF4 expression in the small intestine of CD patients, leading to an impaired differentiation and maturation of goblet cells [12]. Recently, a subset of deregulated miRNAs (i.e., miR-31-5p, miR-192-3p, miR-194-5p, miR-551a, miR-551b-5p, miR-638 and miR-1290) was identified in CD patients with different clinical phenotypes compared with non-CD subjects, and in particular, it was reported that miR-192-3p levels were modulated by gliadin peptides in vitro [13]. Furthermore, it was documented that chemokine C-X-C motif ligand 2 (CXCL2) and NOD2 showed significantly increased mRNA and protein levels in Marsh 3C CD patients and a significant inverse correlation with the regulatory miR-192-5p. In addition, alterations in CXCL2 and NOD2, FOXP3, miR-192-5p and miR-31-5p expression were triggered by gliadin exposure in CD patients [14].

All eukaryotic cells are able to digest their cytoplasmic content through different processes that undergo the general term autophagy (from the Greek words “self” and “to eat”). Autophagy includes different forms of digestive pathways, namely macroautophagy, microautophagy, chaperone-mediated autophagy and non-canonical autophagy. Generally, the term autophagy refers to macroautophagy: this process depends on specialized autophagy-related proteins (Atg) and is able to digest different targets, such as organelles, large aggregates of proteins and microorganisms [15]. Autophagy plays also a key role in direct microorganism and virus clearance, in control of inflammation through the inhibition of the inflammasome, antigen presentation, T cell homeostasis and secretion of immune mediators [16]. Autophagy impairment is crucial in several diseases, in particular proteinopathies, such as Alzheimer’s [17], Parkinson’s [18] and Huntington’s [19]. Autophagy plays also a crucial role in the pathogenesis of several inflammatory diseases: in particular, genetic polymorphisms in autophagy regulatory genes *ATG16L* and *IRGM* conferred a strong predisposition to Crohn’s disease development [20]. Furthermore, SNPs within the autophagy-regulatory *ULK1* gene have been identified to increase susceptibility to Crohn’s disease [21]. On the other hand, limited knowledge is available on the role of the effect of the modulation of the autophagy process in the CD context.

To this purpose, the expression of two main executor genes involved in the canonical autophagy process (i.e., *ATG7* and *BECN1*) and their negative confirmed regulators, respectively miR-17 and miR-30, were investigated in a cohort of pediatric CD patients. Atg7 protein has different functions in autophagosomal biogenesis, acting primary as an E1-like ligase, which conjugates Atg5 to Atg12, a necessary step for the formation of a functional autophagosome. In addition, Atg7 converts Atg8 (LC3) from an immature, cytosolic form to a mature autophagosomal membrane protein by adding a phosphatidylethanolamine group. Genetic deletion of *ATG7* causes an overt loss of autophagy, proving that the gene product is necessary for some autophagic functions [22].

*BECN1* was the first identified autophagy gene [23], and it encodes a protein, Beclin 1, interacting with several cofactors (Atg14L, UVRAG, Bif-1, Rubicon, Ambra1, HMGB1, nPIST, VMP1, SLAM, IP_3_R, PINK and survivin) to regulate in turn the lipid kinase Vps-34 protein and promote the formation of Beclin 1-Vps34-Vps15 core complexes, thereby inducing autophagy. Furthermore, Beclin 1 coordinately regulates the autophagy and membrane trafficking involved in several physiological and pathological processes [24]. Beclin 1 knockout mice die in early embryogenesis, showing that autophagy is clearly necessary for mammalian survival [25].

miR-17 has been identified as the first miRNA with oncogenic potential and therefore named “oncomiR-1” [26]. It is strongly expressed in embryonic stem cells and has essential roles in vital processes like cell cycle regulation, proliferation and apoptosis, representing a promising biomarker and a therapeutic target tool [27]. The importance of miR-17 for fundamental biological processes is underscored by the fact that miR17-deficient mice are neonatally lethal. Recently, miR-17 was identified in the context of aging, turning out to be the first “longevimiR”, extending the lifespan of transgenic miR-17-overexpressing mice [28]. Several studies reported the specific role of miR-17 in modulating the autophagy process in different contexts [29,30,31,32]. The other investigated microRNA, miR-30a, is a member of the miR-30 family [33]. Dysregulation of miR-30a was correlated with several types of malignant tumors [34]. In most of the cancers, miR-30a functions as a tumor suppressor by regulating the corresponding target genes [35]. Noticeably, miR-30a was involved in the regulation of the Beclin 1-mediated autophagy process in different cells [36,37,38,39].

In this paper, novel potential molecular markers (i.e., autophagy-related genes and their regulatory miRNAs) were primary investigated in blood and in intestinal biopsies of pediatric patients with active CD compared with non-CD subjects matched for age and sex, highlighting an advantage for CD diagnosis. In addition, we reported using an in vitro model that the modulation of a specific miRNA’s expression can induce differences in the cellular autophagic status.

## 2. Results

### 2.1. Identification of Autophagy Genes/miRNAs Associated with CD

**Figure 1:**
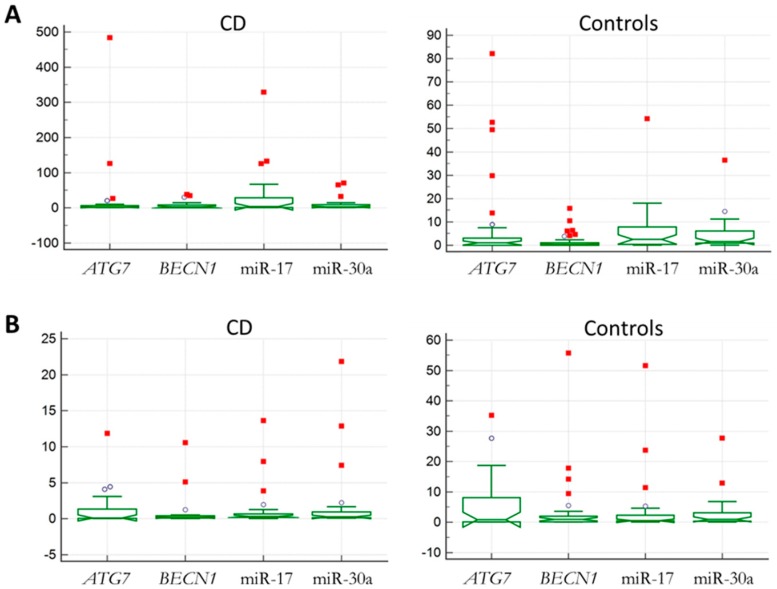
Expression profiles of autophagy-related genes and miRNAs in the investigated tissues. Notched box-and-whisker plot of the expression values obtained by real-time PCR from blood (**A**) or intestinal biopsies (**B**) of celiac disease (CD) patients and controls. In the *Y* axes, the relative quantitative expression (RQ) values are reported. Outliers (**circles**) and far-out values (**squares**) are indicated. Differently, in intestinal biopsies, *ATG7* showed the highest expression profile variability in both groups (Figure 1**B**).

To identify novel molecular markers useful to increase the sensitivity and specificity in pediatric CD patients’ diagnosis, the expression levels of two main autophagy genes and their regulatory miRNAs were analyzed. Specifically, *BECN1* and miR-30a, *ATG7* and miR-17 were investigated. To this purpose, relative quantitative real-time-PCR was performed in blood and in intestinal biopsies derived from exploratory cohorts of pediatric CD patients with active disease compared with controls, as described in the Materials and Methods. Notched box-and-whisker plots of the expression levels of the analyzed markers in both tissues were reported (Figure 1). Among the investigated targets, miR-17 showed the greatest variability in the blood in CD patients and controls (Figure 1A), while, in intestinal biopsies *ATG7* showed the widest range of variations (Figure 1B). In Figure S1, scatterplots highlight the general distribution of the analyzed samples.

To assess the presence of statistically-significant differences in the expression levels between CD patients and controls, the non-parametric Mann–Whitney *U* test was performed. Differences were considered statistically significant when the *p*-value was less than 0.05. In blood, only *BECN1* showed statistically-significant differences between the two analyzed groups (*p*-value = 0.0189). Conversely, in intestinal biopsies, all of the investigated targets showed statistically-significant differences between CD and control subjects (Table 1).

### 2.2. Bioinformatics Performance of the Investigated Targets as Potential CD Biomarkers

To further assess the diagnostic capability of the investigated autophagy-related genes/miRNAs to distinguish between CD and control subjects, the receiver operating characteristics (ROC) analysis was performed (Table 2).

In blood, the *BECN1* ROC curve revealed a fair diagnostic property (AUC = 0.683; *p* = 0.012). The sensitivity and specificity values associated with this curve were 65.22% and 74.29%, respectively. In intestinal biopsies, similar levels within a fair range of sensitivity and specificity were reported for *ATG7, BECN1*, miR-17 and miR-30a, associated with a statistically-significant probability of CD condition.

**Figure 2:**
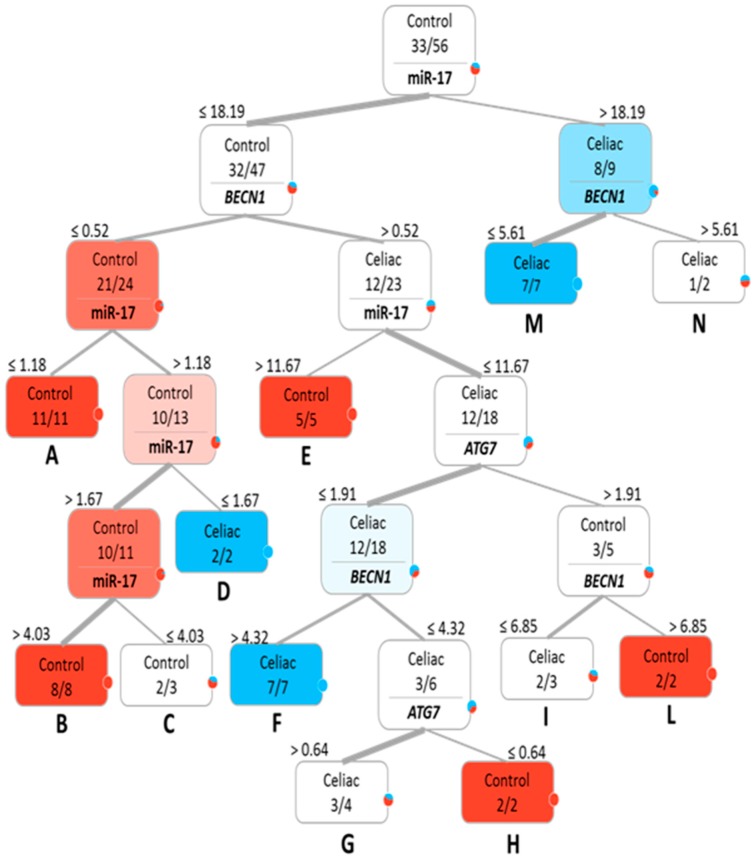
Classification tree obtained using the relative quantitative expression values (numbers over each box) of the analyzed genes (*ATG7* and *BECN1*) and miRNAs (miR-17 and miR-30a) in blood. CD patients are indicated in blue, controls in red. Color tonalities are related to the composition of each subgroup. Resulting subgroups are highlighted by capital letters.

To determine whether the investigated genes/miRNAs were able to effectively distinguish CD and control subjects and to eventually stratify CD patients, a supervised multivariate analysis was conducted. Initially, a classification tree was created on the relative quantitative expression values detected in the blood of CD patients and controls. As reported in Figure 2, two main branches were apically originated by evaluating only miR-17 expression cut-off levels (i.e., inferior/superior of an 18.19-fold expression difference compared to the average of the control samples, used as an internal calibrator): the former classified 32/47 (68.09%) subjects as controls, while the latter 8/9 (88.89%) as CD; evaluating the relative contribution of the some of the other investigated targets (i.e., *BECN1* and *ATG7*), 12 additional homogeneously-represented subgroups were obtained, as indicated by capital letters (A–N). In particular, D, F and M subgroups homogeneously classified 16/23 CD patients (69.56%). In detail, Subgroup G was composed of three CD patients and one control, while two CD patients and one control belonged to Subgroup I. Conversely, Subgroups A, B, H and L homogenously classified 28/33 controls (84.85%).

**Figure 3:**
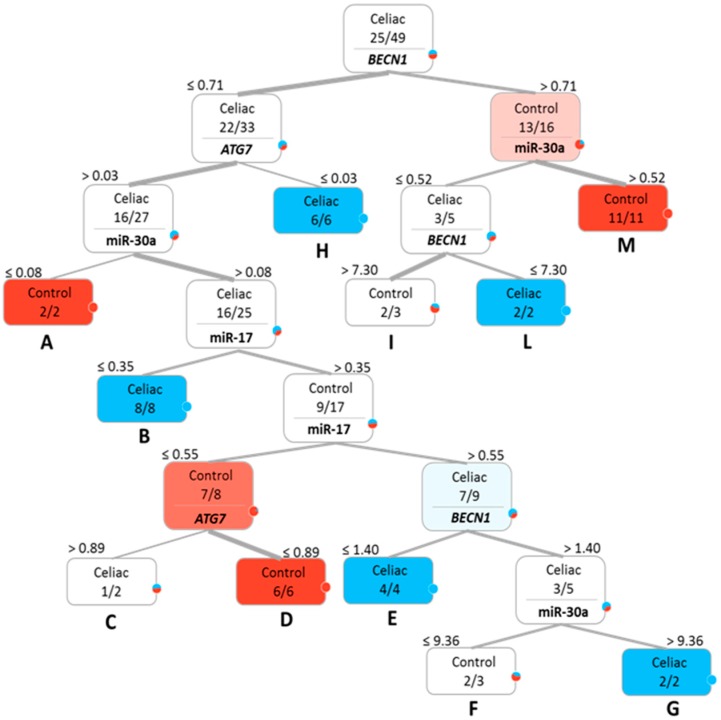
Classification tree obtained using the relative quantitative expression values (numbers over each box) of the analyzed genes (*ATG7* and *BECN1*) and miRNAs (miR-17 and miR-30a) in intestinal biopsies. CD patients are indicated in blue, controls in red. Color tonalities are related to the composition of each subgroup. Resulting subgroups are highlighted by capital letters.

A classification tree was also created for samples derived from intestinal biopsies. As reported in Figure 3, the apical ramification was determined by a *BECN1* expression cut-off value of a 0.71-fold expression difference compared to the average of control subjects (internal calibrator). Then, differently from blood tissue, all of the investigated markers contributed to the next classification roots: in particular, 11 subgroups were obtained, again indicated by the capital letters. Five of them (i.e., B, E, G, H and L) homogeneously classified 22/25 CD patients (88%). Similarly, A, D and M grouped 19/24 controls (79.16%), whereas Subgroups F and I were composed of two controls and one CD patient.

Next, an unsupervised analysis was conducted employing relative gene expression data to test their performance in CD classification. In particular, the classification tree algorithm was assayed to calculate the probability of each subject to be correctly classified in accordance with the initial clinical-based diagnosis. Using the expression levels of the genes/miRNAs detected in the blood, only four subjects (i.e., 10, 29, 36 and 56) were misclassified (Table S1). In the case of intestinal biopsies, only three subjects were misclassified (i.e., 7, 15 and 49), as indicated in Table S2.

**Figure 4:**
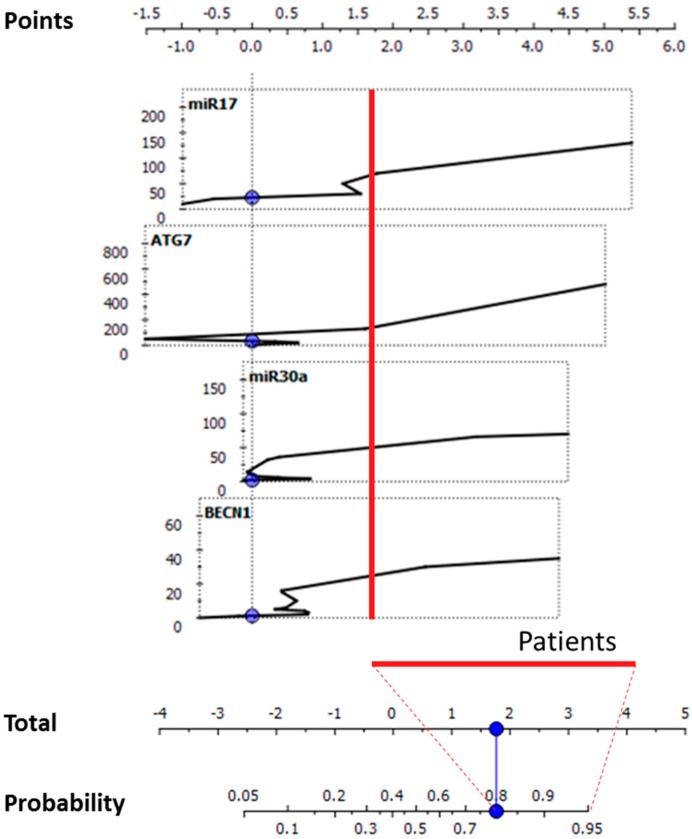
Nomogram tool to predict CD status in the blood specimens. The nomogram (lower graph) was created considering the relative quantitative expression values of the analyzed genes/miRNAs (upper graphs). The red line evidences the portion of the curves (on the right side of each) that confers a high probability (*p* > 0.8) to correctly classify a CD condition.

To determine the relative contribution of each gene/miRNA in improving the CD diagnostic performance, a naive Bayesian nomogram analysis was performed. Relative expression profiles of the investigated targets and their associated probabilities to identify the CD condition were reported (Figure 4).

**Figure 5:**
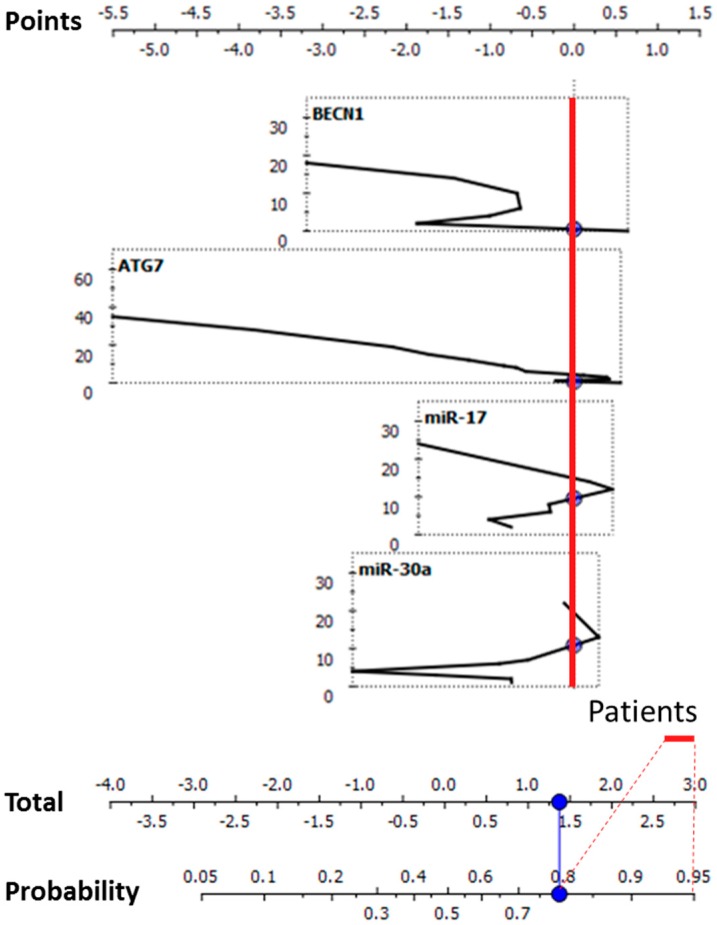
Nomogram tool to predict CD status in the intestinal biopsy specimens. The nomogram (lower graph) was created considering the relative quantitative expression values of the analyzed genes/miRNAs (upper graphs). The red line evidences the portion of the curves (on the right side of each) that confers a high probability (*p* > 0.8) to correctly classify a CD condition.

The expression profiles were represented according to their relative positive influence in determining an increased probability of CD identification, specifically set on *p* > 0.8. In blood samples, as reported in Figure 4, the expression trends were nearly monotonic. An increase in the expression levels of one or more of these markers was therefore nearly proportional to the increase in probability of a correct CD identification (right portions of the curves defined by the red vertical line, corresponding to the highlighted portion of the nomogram of probability). As shown in Figure 5, in intestinal biopsies, *ATG7* showed an opposite nearly-monotonic trend compared to the blood tissue. Differently, *BECN1* and the investigated miRNAs exhibited very complex curves, with higher scores of the defined probability (*p* > 0.8) associated with the CD status in correspondence with low relative expression values.

### 2.3. *In Vitro* Modulation of miR-17 Affects Autophagic Status

**Figure 6:**
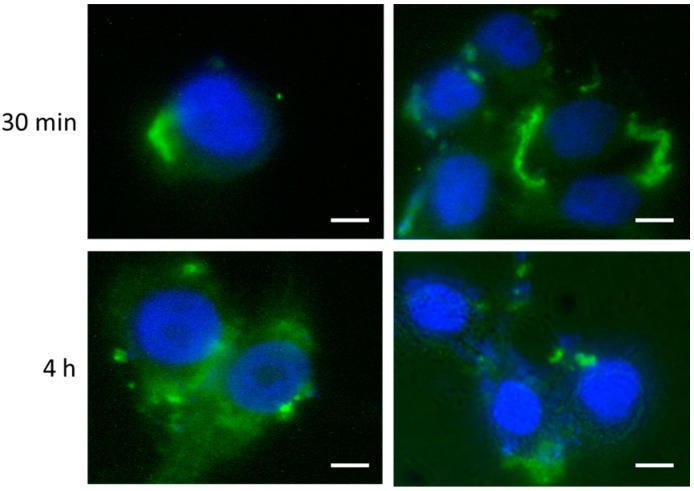
Immunofluorescent detection of digested gliadin uptake in Caco-2 cells. Caco-2 cells were fixed at different times after digested gliadin administration (i.e., 30 min and 4 h). Coverslips were then incubated with anti-gliadin primary antibody followed by a secondary AlexaFluor 488 antibody. Nuclei are stained with DAPI. Size bars are 10 µm.

Next, Caco-2 cells were employed to mimic the induction of a celiac-like condition by direct digested gliadin administration to the cells. Gliadin peptides were previously subjected to a partial enzymatic digestion as described [40]. Digested gliadin uptake was firstly assayed by indirect immunofluorescence (Figure 6).

**Figure 7:**
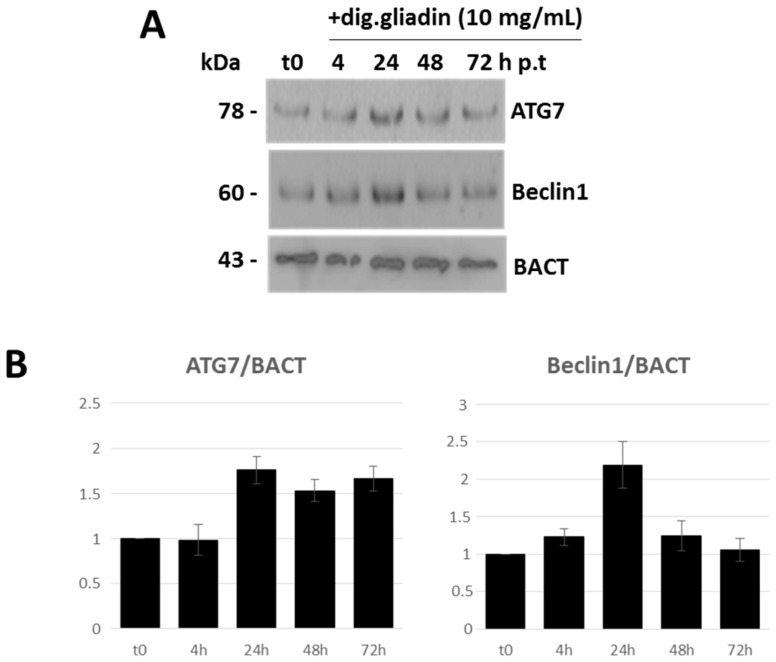
Immunoblotting analysis of autophagy proteins after digested gliadin administration in Caco-2 cells. (**A**) Caco-2 cells were incubated with digested gliadin (10 mg/mL) for different times. Then, proteins were extracted and assayed for expression of Atg7 and Beclin 1 proteins. Molecular weights (kDa) are reported; (**B**) Densitometric analysis was obtained after normalization with BACT expression and referred to untreated (t0) cells. p.t., post-treatment.

As expected, Caco-2 cells rapidly bound digested gliadin at their membranes (soon after 30 min) and finally (from 4 h) internalized a large amount of the extracellular protein content. Then, at different times after digested gliadin administration (i.e., t0, 4, 24, 48 and 72 h post-treatment (p.t.)), total RNA from Caco-2 cells was extracted, quantified and subjected to relative expression analysis by real-time PCR, specifically for miR-17, miR-30a, *ATG7* and *BECN1*. After normalization, miR-17 and miR-30a displayed downregulated expression trends, while, as a direct consequence, *ATG7* and *BECN1* were upregulated. In particular, *ATG7* and its negative regulator miR-17 showed more marked and functionally-correlated differences (Figure S2). Immunoblotting analysis confirmed the induced increase in Atg7 and Beclin 1 protein expression, following digested gliadin administration, again more relevant for the former protein (Figure 7).

**Figure 8:**
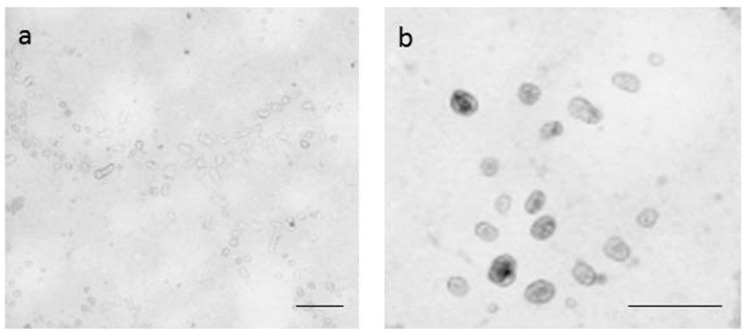
Electron microscopy evaluation of exosomes isolated from T98G cells at different magnification (**a**,**b**). Exosomes were isolated from exosome depleted FBS-containing medium of T98G growing cells at about 80% of confluence from a 90-mm cell plate and visualized by transmission electron microscopy. The bar size is 200 nm.

Next, a modulation of miR-17 expression, to affect the Atg7 protein profile, was performed in Caco-2 cells, using exosomes as cargo vehicles of the specific miRNA. Exosomes were primary isolated from different human cancer cells (HeLa, Caco-2, T98G) using Total Exosome Isolation (from cell culture media) as described in the Materials and Methods. Total exosomal RNAs were isolated and assayed by Real-time PCR for miR-17 expression. Exosomes showing less intra-organelle content of miR-17 were employed for the next experiments; specifically, T98G-derived exosomes highlighted the most reduced content of miR-17 (real-time PCR quantitative relative miR-17 expression, using HeLa miR-17 levels as the internal calibrator, were: HeLa = 1 ± 0.11; Caco-2 = 0.77 ± 0.21; T98G = 0.36 ± 0.19). T98G-derived exosomes were also visualized by electron microscope (Figure 8), showing a relative overall maintenance of their integrity after isolation procedures.

**Figure 9:**
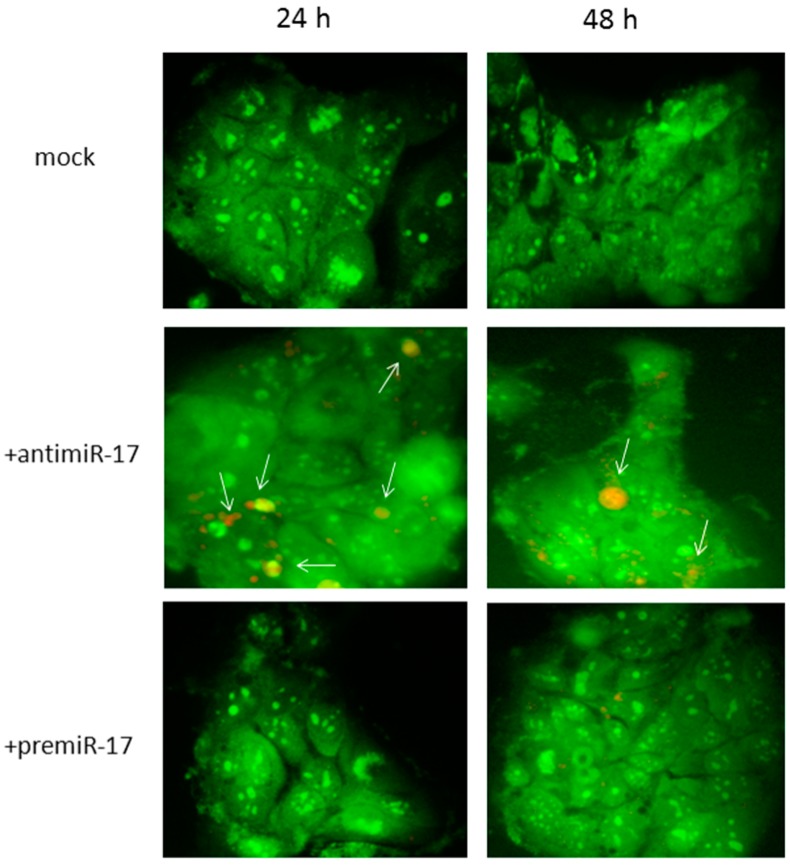
Fluorescent evaluation of autophagy vesicles in Caco-2 cells after differential exosome uptake. Exosomes isolated from T98G cells were electroporated with antimiR-17 or premiR-17 molecules (2.5 µM each) and administered or not (mock) to growing Caco-2 cells. After 24–48 h post-administration, cells were then incubated with acridine orange dye (200 µg/mL, for 5 min at 37 °C) and then visualized by inverted fluorescent microscope evaluation (40× magnification). Arrows indicate large acidic vesicles, likely autophagosomes.

These exosomes were then subjected to specific electroporation conditions to force mimic-miR17 or antagomir-miR17 entrance (both at 2.5 µM). Relative quantitative real-time PCR experiments were performed to evaluate the efficiency of the delivery of miRNA moieties by electroporation into T98G-derived exosomes, evaluating the resulting levels of miR-17: according to the employed electroporation conditions, miR-17 levels were increased to an (18.36 ± 0.26)-fold difference, compared to the endogenous miR-17 levels of non-electroporated T98G exosomes. Finally, these exosomes were separately administered to growing Caco-2 cells. After 24 and 48 h p.t., the autophagic status of the cells (i.e., the presence of large acidic vesicles as autophagosomes) was evaluated by acridine orange fluorescent staining. As a result, the administration of anti-miR17-enriched exosomes resulted in a clear increase of red acidic vesicular organelles (AVOs) characterized by internal undigested content: this pattern, suggestive of autophagy activation, was markedly different compared to pre-miR17 exosomes or to untreated cells (Figure 9). To quantify these results, red-acridine orange positive vesicles (AVOs > 1 μm^2^) were analyzed in number and shape (area) using the Autocounter tool as we originally reported [41]. Comparing to untreated cells, antimiR-17 treatment resulted in an increase of 72% and 85% in the number and area of vesicles, compared to premiR-17 (22% and 35%), respectively.

## 3. Discussion

In this study, novel biomarkers related to the autophagy process were investigated in blood and in intestinal biopsies of pediatric patients with active celiac disease (CD) compared with non-CD subjects matched for age and sex. CD is an immune-mediated systemic disorder of the small intestine elicited by gluten and related prolamins in genetically-susceptible individuals and characterized by the presence of different clinical manifestations [1,2,3,42].

As already stated, the autophagy process was primarily investigated due to its relative importance in host defense against intra- and extra-cellular pathogens, metabolic syndromes, immune cell homeostasis, antigen processing and presentation, as well as for its role in autoimmune disorders [43]. Our study was conducted on a cohort of pediatric CD patients and control subjects, clinically classified as described in the Material and Methods. All of the samples were analyzed through real-time PCR for the relative expression of two essential autophagic genes (*ATG7* and *BECN1*) and their functionally-related miRNAs (miR-17 and miR-30a).

As introduced before, Beclin 1 and Atg7 are key proteins implicated in autophagy, respectively in the nucleation phase and in autophagosome elongation [15]. However, among the *ATG* genes, *ATG6* or *BECN1* is relatively unique in its not being “autophagy-specific”. In fact, recent findings indicated that Beclin 1 is a multifaceted protein that governs additional processes, such as endocytosis, phagocytosis and cytokinesis. More generally, Beclin 1 is involved in development, differentiation, stress adaptation, inflammation, tumorigenesis, aging and cell death [44]. All of these processes have in common the regulation of membrane flux and their specific corresponding controls, where the autophagic and non-autophagic functions of Beclin 1 can be determined by the binding of specific interactors that define its subcellular localization and trafficking [45]. On the other hand, it is widely documented that the expression levels of Beclin 1 are tightly controlled, since many diseases are associated with Beclin 1 malfunction or deficiency, and therefore, Beclin 1 is considered a valuable target for the treatment of distinct pathologies [46]. Atg7 is a protein playing a central role in the canonical autophagy process, specifically in the elongation of the autophagosome membranes. Recent works have suggested that autophagy can occur using multiple variant mechanisms, bypassing seemingly essential proteins, including Atg7. In fact, a dispensable function of Atg7 was reported, replaced by TRIM31 in Chron’s disease [47]; other authors suggested alternative routes to macroautophagy without a significant contribution of Atg5 and Atg7 [48]. Furthermore, mice models of experimentally-induced colitis with intestinal epithelial deletion of Atg7 showed reduced size of granules and decreased levels of lysozyme in Paneth cells, suggesting that this protein was dispensable for gut immune homeostasis and had no effect on susceptibility [49]. Conversely, other autophagy-related genes (*ATG16L1* and *IRGM*) seemed to play important roles for autophagy in maintaining intestinal homeostasis, and the overall dysfunction of autophagy resulted in being a major risk factor for the onset of chronic intestinal inflammation [20].

In general, alternative mechanisms of autophagy are growing in which Atg proteins appear to be dispensable under some circumstances. This might reflect a still unraveled complexity of the autophagy process. We have also to consider that, while much of our understanding of autophagy mechanisms comes from in vitro systems using either yeast or mammalian cell lines, the significance of autophagy in pathology is mostly derived from experimental animal models. Using animals to model defective autophagy mechanisms may, however, introduce an additional layer of complexity in our attempts to understand cell type-specific defects in autophagy [50].

Among all of the miRNAs that play a role in autophagy regulation, miR-30a and miR-17 were selected because of their experimental validation as negative regulators of defined molecular steps within the autophagic process. Particularly, miR-30a negatively regulated *BECN1* expression resulting in a decrease of the autophagy activity. Moreover, it was demonstrated that treatment of different cancer cell lines with the miR-30a mimic and antagomir molecules could respectively decrease and increase *BECN1* expression [51]. A similar approach was used on T98G glioblastoma cells to demonstrate that miR-17 negatively regulated *ATG7* expression, thus contributing to an overall downregulation of the autophagic process [29].

In relation to the clinically-established diagnostic criteria in CD [3], the tissues assayed in the present study were blood and intestinal biopsies. In the former tissue, the expression levels of *BECN1* differed significantly between CD patients and controls. The area under the ROC curve confirmed the levels of expression of *BECN1* as a marker that can fairly distinguish between the two diagnostic groups. The differences in the levels of expression were confirmed by the ROC analyses, and the area under the ROC curve highlighted the capability to significantly cluster patients and controls. Differently, considering intestinal biopsies, the expression of all investigated molecular targets gave statistically-significant performance in distinguishing between patients affected by CD. To determine whether the investigated genes/miRNAs were able to effectively stratify the CD patients, a supervised multivariate analysis was conducted. Classification trees were used based on the decision tree learning model [52]. In blood, miR-17 constituted the first decision node of the tree, determining together with *BECN1* expression levels the first subgroup of CD patients. The other subgroups are determined by the algorithm taking into account all of the targets excluding miR-30a. Altogether, CD patients homogeneously classified were 16/23 (69.50%), suggesting that blood guaranteed a discreet performance in CD patients’ stratification. The classification tree obtained for intestinal biopsies homogeneously classified 22/25 CD patients (88%) into five subgroups using the expression values of *BECN1* (as the apical node), *ATG7* and their negative regulators, miR-17 and miR-30a. Subsequently, the ability of the algorithm to correctly classify all of the subjects according to the initial clinical-based diagnosis was evaluated. The number of subjects correctly classified was 52/56 (92.86%) in the case of blood and 46/49 (93.88%) analyzing the intestinal biopsy specimens. Then, nomograms were created for both investigated tissues using the Orange software [53] to evaluate the relative contribution of each gene/miRNA in determining an increase in the CD diagnostic probability performance. A nomogram is a dynamic supervised statistical tool used to analyze continuous and categorical variables together, adopting a heuristic problem solving approach [54]. The algorithm calculates for each continuous variable a score and a probability associated with the disease/physiological state. This tool was used also in other pathological contexts, i.e., glioblastoma [55,56] in prostate, breast, ovarian, renal cell carcinoma and melanoma to stratify patients and to find specific molecular signatures [57]. In blood, a high rate of CD patients’ probability was primarily determined by the increase of miR-17, followed by *ATG7*, miR-30a and *BECN1* expression levels. Although the Mann–Whitney *U* test and ROC analyses did not identify any miRNAs as able to distinguish among CD patients and controls, according to the blood-predicted nomogram, miR-17 played a relevant role in increasing the CD likelihood. In intestinal biopsies, the originated nomogram pointed to a decreased expression trend for *ATG7* associated with CD diagnostic probability, while *BECN1* and the two miRNAs exhibited complex and non-linear expression profiles.

**Figure 10:**
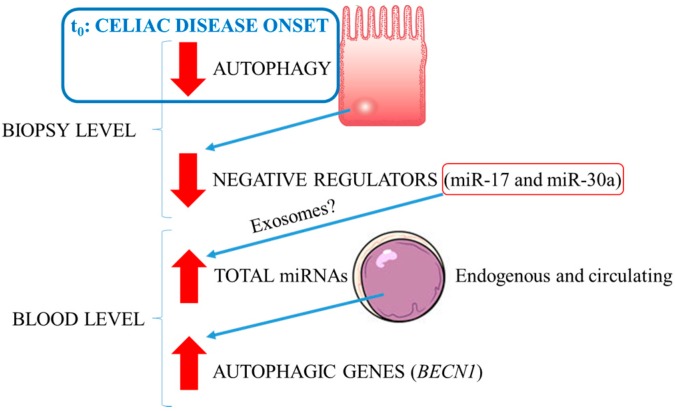
Interpretative model of autophagy modulation as inferred by *ATG*s’ and miRNAs’ expression profiles in the intestine (biopsy level) and in the blood of CD patients. Red arrows define autophagy status (i.e. down-regulation or up-regulation).

Based on the described evidence, a parsimonious interpretation model of the expression results is proposed (Figure 10). 

As shown, CD onset would correspond with an autophagy impairment in epithelial cells of the intestine, as revealed by the *ATG7* and *BECN1* downregulation nomogram trends. The decrease in the expression of miR-17 and miR-30a could be explained as the result of a compensatory mechanism triggered by this pathological loss of homeostasis in the autophagic process, in order to counteract the downregulation of the autophagy-related one. The miR-30a and miR-17 decreasing expression trend could be further mediated by the release of these miRNAs as a cargo within exosomes, thus in part explaining the high levels revealed by blood nomograms. Finally, in this tissue, the increase of *ATG7* and *BECN1* relative expression could be due to another compensatory process to maintain autophagy homeostasis after the exosome-mediated release of miR-17 and miR-30a from the epithelial cells.

Lastly, the in vitro results using the Caco-2 cell model, widely adopted in CD studies [58,59] and challenged with a direct digested gliadin administration, provided a possible mechanistic view of the regulatory effects of miRNAs, specifically miR-17, on the expression of a key autophagic gene as *ATG7*. Similar to our previous results [29], we showed that even only modulating intracellular miR-17 levels, this can significantly alter the autophagic status of the cells. In the results herein reported, anti-miR17-enriched exosomes, obtained through a specific electroporation protocol, induced a prominent increase in acridine orange red positive vesicles. These vesicles had an acidic content, due to the spectral property of the fluorescent dye [60], and additionally, microscope visualization indicated the presence of undigested materials, typical of autophagosomes. We can also speculate that the activation of the autophagy process might counteract the gliadin cellular toxicity, leading to a possible digestion of these exogenous proteins. With this regard, additional in vitro experiments to confirm this hypothesis are ongoing (Comincini, personal communication).

Certainly, these results need further investigations to confirm the diagnostic power of the investigated autophagic markers. In particular, additional autophagy-related genes and their regulatory miRNAs should be included in a larger cohort of CD patients and controls to strengthen the results obtained in this study, since the statistical resolution of data mining and machine learning analyses, i.e., classification trees and nomograms, depends on the size of the analyzed cohort. Of note, it was demonstrated that the autophagy process could have a functional role within this pathological condition. Indeed, expression levels of key autophagic genes (*ATG7* and *BECN1*) and their regulatory miRNAs (miR-17 and miR-30a) in the blood and intestinal biopsies can give molecular signatures useful to classify and stratify CD patients. This positive feature made these genes and miRNAs interesting candidates as novel non-invasive biomarkers in the CD field. Furthermore, according to the interpretative model and with the in vitro results, exosomes content might be deeply investigated, thus determining a profile of endogenous and circulating miRNAs in the blood of CD patients, with the potentiality to regulate in different areas important cellular processes, like autophagy.

## 4. Materials and Methods

### 4.1. Investigated Celiac Patients and Controls

Pediatric patients were enrolled in collaboration with the Pediatric Auxology Unit and the Pediatric Surgery Unit of the Fondazione Istituto di Ricovero e Cura a Carattere Scientifico (IRCCS) Policlinico San Matteo (Pavia, Italy) over the last two years. This study (project identification code: 31140/2014, date of approval: 16 December 2013) was approved by the Ethical Committee “Comitato Etico Area di Pavia” (Pavia, Italy), and written informed consent was obtained from the parents of all of the subjects. The cohort of subjects submitted to CD screening was divided into two groups, as described in Table S3. CD patients were enrolled for the concomitant presence of symptoms (anemia, short stature, gastrointestinal symptoms given by malabsorption). Positive serological tests and histological analysis of the biopsies confirmed the CD diagnosis. Control patients were referred to clinicians for other reasons not related to CD. Some of them underwent upper gastrointestinal endoscopy for other clinical purposes, such as gastroesophageal reflux, esophageal varices, gastritis or *H. pylori* infection. The exclusion criteria were dysmorphic syndromes, chromosomal abnormalities and chronic conditions causing growth retardation. A total of 105 specimens were collected, specifically 56 blood samples and 49 intestinal biopsies. Blood samples were collected from 33 controls and 23 untreated CD patients, whereas biopsies were collected from 24 controls and 25 untreated CD patients, divided into the Marsh 3B and 3C pathological grading. Blood (3 mL for each sample) was collected in EDTA tubes, whereas duodenal specimens (5–10 mm^3^ for each biopsy) were collected in 1.5 mL of TRIzol reagent (Invitrogen, Carlsbad, CA, USA). Both types of tissues were stored at −80 °C until RNA extraction.

### 4.2. Real-Time PCR Expression Analysis

Total RNA was extracted from blood and biopsies tissues with TRIzol reagent according to the manufacturer’s instructions. Before RNA extraction, biopsies were homogenized with Tissue Ruptor (Qiagen, Hilden, Germany). RNA quantification was performed using the Quant-it RNA Assay (Invitrogen). miRNAs’ cDNA was obtained using a sequence-specific hairpin-primer and amplified using the TaqMan MicroRNA Assays kit (Applied Biosystems, Foster City, CA, USA). *ATG7* and *BECN1* cDNA were obtained using random hexamers primers (Applied Byosystems, Foster City, CA, USA ) as reported in Comincini et al. [29]. *ATG7* (TaqMan Assay ID Hs00893766_m1), *BECN1* (TaqMan Assay ID Hs01007018_m1), miR-17 (TaqMan Assay ID 478447_mir) and miR-30a (TaqMan Assay ID 479448_mir) were amplified with TaqMan Universal Master Mix II, no UNG (Applied Biosystems). Real-time PCR was performed using 5 ng of each cDNA and using the MiniOpticon Real-Time PCR System (Bio-Rad, Hercules, CA, USA) platform. Samples were analyzed in duplicate and subjected to 40 amplification cycles of 95 °C for 15 min and 60 °C for 1 min. All data were normalized to *ACTB* (TaqMan Assay ID Hs01060665_g1) and relative quantification with the 2^−ΔΔ*C*t^ method [61] was employed to calculate relative changes in gene expression using an internal calibrator (control sample) obtained calculating the mean of all of the controls. All of the relative quantification values were checked for the presence of outliers and far-out values. The samples with relative quantification values very far from the mean were analyzed a second time, and the outliers were verified as real. As the deletion of outliers is a controversial practice especially for relatively small cohorts, all of the statistical analyses were performed including also the samples with outliers and far-out values.

### 4.3. Digested Gliadin *In Vitro* Assay

Caco-2 cells from the American Type Culture Collection (ATCC) were cultured and maintained in DMEM (Euroclone, Milano, Italy) supplemented with 10% FBS, 100 units/mL penicillin, 0.1 mg/mL streptomycin and 1% l-glutamine and kept at 37 °C in a 5% CO_2_ air atmosphere. Gliadin from wheat (Sigma, St. Louis, MO, USA) was digested as described [40] to a final concentration of 1 g/mL. In detail, gliadin was firstly dissolved in 500 mL 0.2 N HCl for 2 h at 37 °C with 1 g of pepsin (Sigma). The resultant peptic digest was further digested by the addition of 1 g trypsin (Sigma), after pH adjustment to 7.4 using 2 N NaOH, and the solution was incubated at 37 °C for 4 h with vigorous agitation. Finally, the mixture was boiled to inactivate enzymes for 30 min and stored at −20 °C. For in vitro assays, digested gliadin (10 mg/mL) was firstly administered directly to Caco-2 cells in suspensions (about (1–1.5) × 10^6^) for 30 min at 37 °C, and then cells were distributed in multiwell 12-wells plates for different time intervals. 

### 4.4. Immunofluorescence and Immunoblotting Analysis

Immunofluorescence assays were performed similarly to [62]. In detail, primary antibody anti-gliadin (Abcam, Cambridge, UK) was diluted 1:30 and used to trace the gliadin uptake and internalization into vesicles. Species-specific Alexa Fluor 488 secondary conjugated antibody (1:100) (Invitrogen) was then employed. Cells cultured on coverslips were washed three times with PBS and fixed with 4% paraformaldehyde-PBS, pH 7.4, for 15 min at room temperature. Cells were incubated for 1 h with primary antibody diluted in PBS + 5% non-fat milk powder (*w/v*). Cells were then washed with PBS and incubated for 1 h with secondary antibody in PBS + 5% non-fat milk powder. Coverslips were then washed three times with PBS and treated with ProLong Gold antifade reagent with DAPI (Invitrogen) according to the manufacturer’s instructions and finally mounted onto glass slides. Fluorescence signals were detected using a fluorescence light inverted microscope (Nikon Eclipse TS100, Tokyo, Japan) with a 100× oil immersion objective. Western blot analysis was performed using procedures previously described [63]. The following primary antibodies directed against the antigens were used: Atg7 and Beclin 1 (1:1000, rabbit polyclonal, Cell Signaling, Danvers, MA, USA), while *ACTB* (1:5000, mouse monoclonal, Cell Signaling) was used as the loading control in the Western blots, as already reported [64].

### 4.5. Exosome Isolation, Visualization and Electroporation

Exosomes were isolated using Total Exosome Isolation (from cell culture media) (Invitrogen,) according to the manufacture’s specification. In detail, assayed cells (HeLa, T98G, Caco-2) were grown near to their confluence in 10 mL of complete DMEM medium supplemented with 10% of Exosome Depleted FBS Qualified Origin (Invitrogen). Culture media were collected, and 5 mL of Total Exosome Isolation reagent for Cell Culture were then added to the media and incubated at 4 °C for 20 h. Then, exosomes were pelleted at 10,000 rpm for 60 min at 4 °C and finally resuspended in 0.5 mL of sterile ice-cold Dulbecco’s PBS. Half of each exosome’s preparations were used for RNA extraction using TRIzol reagent. RNA was quantified (typically 2–6 ng for each isolation), subjected to cDNA synthesis specific for miR-17 or miR-30a and then assayed in relative quantitative expression by real-time PCR, using the above-reported conditions. Exosomes from T98G cells, showing the lowest miR-17 expression levels, were selected for further experiments directly to modulate their intravesicular content of miR-17 levels; these exosomes were then visualized by transmission electron microscopy. In detail, 20 microliter drops of the isolated exosomes in D-PBS were placed on a Parafilm (Sigma) sheet, and a 300-mesh nickel grid (covered with a Formvar-carbon film) was floated onto the drops and allowed to stay for 5 min. The grids were rapidly blotted with filter paper and negatively stained with a 2% phosphotungstic acid solution, pH 7.0, for 60 s, blotted on paper and observed directly on a Zeiss EM900 electron microscope (Zeiss, Oberkochen, Germany) operating at 80 kV. Next, 100 microliters of exosomes in D-PBS were electroporated with 2.5 µM of each mimic (premiR-17, Ambion #AM25576), antagomiR (antimiR-17, Ambion #AM17010) or without (mock). For electroporation, the Neon Transfection System (Invitrogen) along with the Neon 100 µL kit (Invitrogen) were used and adopting these physical parameters: voltage = 700 V, width = 10 ms, pulse = 10.

### 4.6. Acridine Orange Staining of Autophagic Vesicles

The direct visualization of acidic vesicles was performed using an acidotropic dye, i.e., acridine orange (excitation/emission: 502/525 nm). Acridine orange was added at the final concentration of 1 µg/mL in Caco-2 cultures 10 min before inverted microscope fluorescent visualizations. As already documented [60], different intracellular vesicles emitted in the red range because of the protonation of the dye caused by their acidic content. Autophagosomes and lysosomes can also be distinguished according to their shape and size since the former are more heterogeneous and bigger in dimension [60]. An inverted fluorescence microscope (Eclipse Nikon TS100) was employed for live cell monitoring using 40× magnification.

### 4.7. Statistical Analysis

The MedCalc software 15.8 (https://www.medcalc.org/) was used to perform the Mann–Whitney *U* test and the receiver operating characteristic (ROC) curve analysis. The Orange data mining software (http://orange.biolab.si/) was used for the nomograms and the classification trees using a naive Bayes learner/classifier. Differences were considered statistically significant when *p* < 0.05.

## 5. Conclusion

This study demonstrated that autophagy investigation could be promising at different levels in celiac disease (CD) research. Indeed, expression levels of key autophagic genes (*ATG7* and *BECN1*) and miRNAs (miR-17 and miR-30a) in the peripheral blood and intestinal biopsies can fairly distinguish between CD patients and controls. This positive feature made these genes and miRNAs interesting candidates as novel non-invasive biomarkers in CD field, thus recommending further investigations on larger cohorts to assess their potential diagnostic power and to find other interesting candidate as biomarkers. Moreover, the present study highlighted a possible role of these autophagy targets in CD patients’ stratification, especially at the bioptic level. In addition, the creation of nomograms as supporting tools to assist in CD diagnosis, particularly in potential CD, seems to be clinically relevant. Lastly, in vitro experiments, showed the possibility to increase autophagy through the modulation of miR-17 levels using anti-miR17-enriched exosomes, with the aim to digest gliadin thus counteracting its toxicity. In conclusion, it was demonstrated that genes and miRNAs involved in autophagy might have a potential diagnostic utility for CD and for the creation of clinical predictive tools. Moreover, deeper investigation on within this process and its modulation could be a promising strategy in developing new CD treatments. Finally, although the complexity of this disease and its clinical management, this study might present new interesting perspectives in CD diagnosis and therapy research.

## Figures and Tables

**Table 1:** Association of autophagy-related genes’ and miRNAs’ relative expression with CD status.

Tissue	Gene/miRNA	*Z*-Score	*p*-Value
Blood	*ATG7*	2.13	0.2585
	*BECN1*	2.34	0.0189 *
	miR-17	1.13	0.2557
	miR-30a	1.51	1.1310
Intestinal biopsy	*ATG7*	2.41	0.0159 *
	*BECN1*	2.48	0.0129 *
	miR-17	2.09	0.0365 *
	miR-30a	2.16	0.0302 *

*Z*-scores and *p*-values obtained with the Mann–Whitney *U* test. The asterisks indicate a statistically-significant association with CD (*p*-value < 0.05).

**Table 2:** ROC curve analysis of the association of autophagy-related genes and miRNAs relative expression with CD status.

Tissue	Gene/miRNA	AUC	C.I. 95%	*p*-Value	Sensitivity	Specificity
Blood	*ATG7*	0.603	0.46–0.73	0.1723	78.26	47.06
	*BECN1*	0.683	0.54–0.79	0.012 *	65.22	74.29
	miR-17	0.605	0.46–0.73	0.1822	34.78	97.06
	miR-30a	0.632	0.49–0.75	0.0754	56.52	67.65
Intestinal biopsies	*ATG7*	0.697	0.55–0.81	0.007 *	64.00	69.23
	*BECN1*	0.703	0.55–0.82	0.0068 *	88.00	57.69
	miR-17	0.671	0.52–0.79	0.0274 *	56.00	84.62
	miR-30a	0.677	0.53–0.80	0.0238 *	72.00	69.23

Area under the curve (AUC), confidence intervals (C.I., at 95%), sensitivity and specificity percentage values are reported. The asterisks indicate a statistically-significant association with CD status (*p*-value <0.05).

## Supplementary Materials

Supplementary materials can be found at www.mdpi.com/1422-0067/18/2/391/s1.
