# Supplementary material for: Identification of Autophagy-Related Genes and Their Regulatory miRNAs Associated with Celiac Disease in Children
Source: Int J Mol Sci. 2017 Feb 12;18(2):391. doi: 10.3390/ijms18020391 (PMC5343926; doi:10.3390/ijms18020391)
Supplement: Supplementary file 1 [file ijms-18-00391-s001.pdf]

Supplementary Materials: Identification of Autophagy-Related Genes and Their Regulatory miRNAs Associated with Celiac Disease in Children

Sergio Comincini, Federico Manai, Cristina Meazza, Sara Pagani, Carolina Martinelli, Noemi Pasqua, Gloria Pelizzo, Marco Biggiogera and Mauro Bozzola

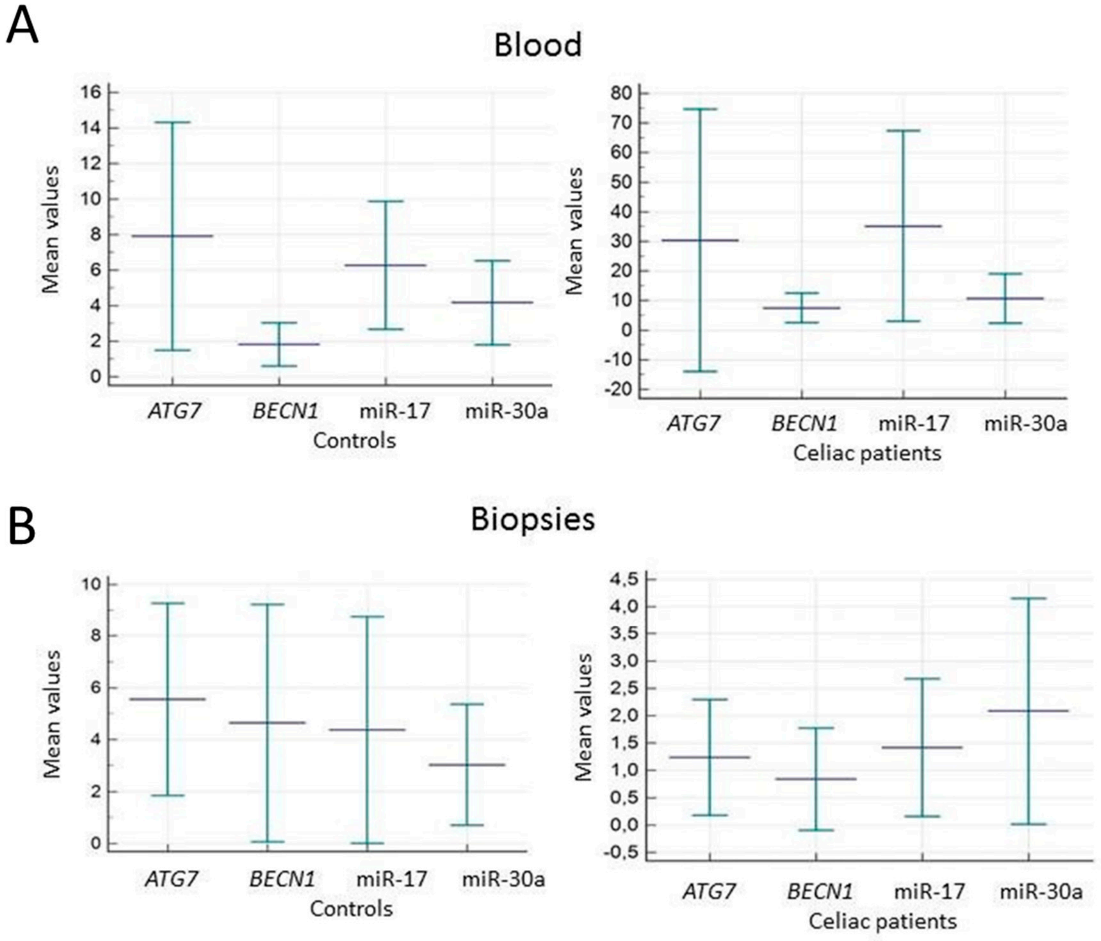

Figure S1. Distribution of the investigated autophagy-related genes (*ATG7* and *BECN1*) and their regulatory miRNAs (miR-17 and miR-30a) in blood (**A**) and biopsies (**B**). The expression values are reported as mean and 95% C.I.

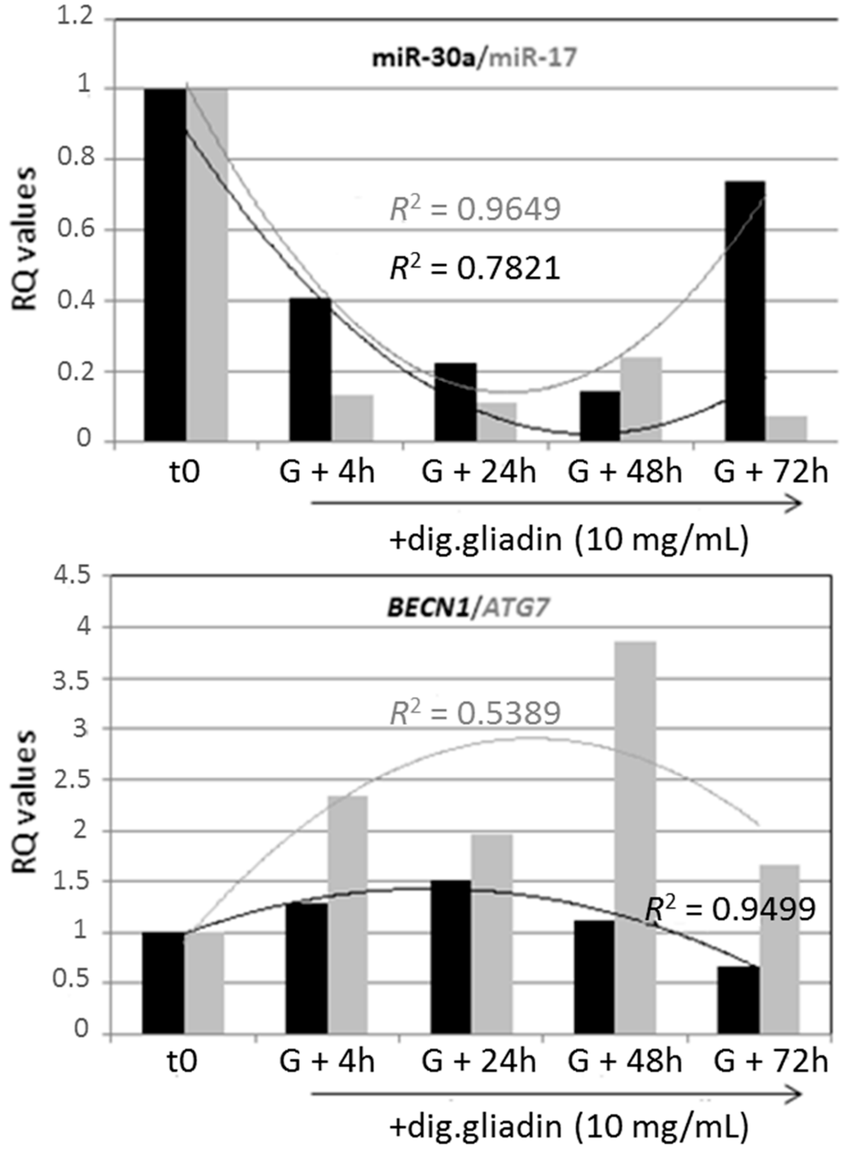

Figure S2. Relative quantitative expression (RQ) of autophagy-related genes (*ATG7* and *BECN1*) and their regulatory miRNAs (miR-17 and miR-30a), following digested gliadin administration at different post-treatment times. Normalization was referred to untreated conditions. Exponential regression trends and their significance (R^2) are reported.

Table S1. Unsupervised analysis of celiac disease classification performance adopting the expression values of the investigated genes/miRNAs in the peripheral blood.

Unsupervised Analysis for Blood Samples			
**Subject**	**Diagnosis**	**CD vs. Control Probability**	**Classification**
1	Celiac disease	1.00 : 0.00	Celiac disease
2	Celiac disease	0.50 : 0.50	Celiac disease
3	Celiac disease	0.67 : 0.33	Celiac disease
4	Celiac disease	1.00 : 0.00	Celiac disease
5	Celiac disease	1.00 : 0.00	Celiac disease
6	Celiac disease	1.00 : 0.00	Celiac disease
7	Celiac disease	0.75 : 0.25	Celiac disease
8	Celiac disease	1.00 : 0.00	Celiac disease
9	Celiac disease	1.00 : 0.00	Celiac disease
10	Celiac disease	0.33 : 0.67	Control *
11	Celiac disease	0.67 : 0.33	Celiac disease
12	Celiac disease	1.00 : 0.00	Celiac disease
13	Celiac disease	1.00 : 0.00	Celiac disease
14	Celiac disease	1.00 : 0.00	Celiac disease
15	Celiac disease	1.00 : 0.00	Celiac disease
16	Celiac disease	0.75 : 0.25	Celiac disease
17	Celiac disease	1.00 : 0.00	Celiac disease
18	Celiac disease	1.00 : 0.00	Celiac disease
19	Celiac disease	0.75 : 0.25	Celiac disease
20	Celiac disease	1.00 : 0.00	Celiac disease
21	Celiac disease	1.00 : 0.00	Celiac disease
22	Celiac disease	1.00 : 0.00	Celiac disease
23	Celiac disease	1.00 : 0.00	Celiac disease
24	Control	0.00 : 1.00	Control
25	Control	0.00 : 1.00	Control
26	Control	0.00 : 1.00	Control
27	Control	0.00 : 1.00	Control
28	Control	0.00 : 1.00	Control
29	Control	0.50 : 0.50	Celiac disease *
30	Control	0.00 : 1.00	Control
31	Control	0.00 : 1.00	Control
32	Control	0.00 : 1.00	Control
33	Control	0.00 : 1.00	Control
34	Control	0.00 : 1.00	Control
35	Control	0.00 : 1.00	Control
36	Control	0.67 : 0.33	Celiac disease *
37	Control	0.00 : 1.00	Control
38	Control	0.00 : 1.00	Control
39	Control	0.00 : 1.00	Control
40	Control	0.00 : 1.00	Control
41	Control	0.33 : 0.67	Control
42	Control	0.00 : 1.00	Control
43	Control	0.33 : 0.67	Control
44	Control	0.00 : 1.00	Control

Table S1. *Cont.*

Unsupervised Analysis for Blood Samples			
**Subject**	**Diagnosis**	**CD vs. Control Probability**	**Classification**
45	Control	0.00 : 1.00	Control
46	Control	0.00 : 1.00	Control
47	Control	0.00 : 1.00	Control
48	Control	0.00 : 1.00	Control
49	Control	0.00 : 1.00	Control
50	Control	0.00 : 1.00	Control
51	Control	0.00 : 1.00	Control
52	Control	0.00 : 1.00	Control
53	Control	0.00 : 1.00	Control
54	Control	0.00 : 1.00	Control
55	Control	0.00 : 1.00	Control
56	Control	0.75 : 0.25	Celiac disease *

Celiac disease vs. control probability was calculated (second column) in order to compare the obtained classification with the diagnosis. Asterisks highlight misclassified subjects.

Table S2. Unsupervised analysis of celiac disease classification performance adopting the expression values of the investigated genes/miRNAs in the intestinal biopsies.

Unsupervised Analysis for Intestinal Biopsies			
**Subject**	**Diagnosis**	**CD vs. Control Probability**	**Classification**
1	Celiac disease	1.00 : 0.00	Celiac disease
2	Celiac disease	1.00 : 0.00	Celiac disease
3	Celiac disease	1.00 : 0.00	Celiac disease
4	Celiac disease	1.00 : 0.00	Celiac disease
5	Celiac disease	1.00 : 0.00	Celiac disease
6	Celiac disease	1.00 : 0.00	Celiac disease
7	Celiac disease	0.33 : 0.67	Control *
8	Celiac disease	1.00 : 0.00	Celiac disease
9	Celiac disease	1.00 : 0.00	Celiac disease
10	Celiac disease	1.00 : 0.00	Celiac disease
11	Celiac disease	1.00 : 0.00	Celiac disease
12	Celiac disease	1.00 : 0.00	Celiac disease
13	Celiac disease	1.00 : 0.00	Celiac disease
14	Celiac disease	1.00 : 0.00	Celiac disease
15	Celiac disease	0.33 : 0.67	Control *
16	Celiac disease	1.00 : 0.00	Celiac disease
17	Celiac disease	1.00 : 0.00	Celiac disease
18	Celiac disease	1.00 : 0.00	Celiac disease
19	Celiac disease	1.00 : 0.00	Celiac disease
20	Celiac disease	1.00 : 0.00	Celiac disease
21	Celiac disease	0.50 : 0.50	Celiac disease
22	Celiac disease	1.00 : 0.00	Celiac disease
23	Celiac disease	1.00 : 0.00	Celiac disease
24	Celiac disease	1.00 : 0.00	Celiac disease
25	Celiac disease	1.00 : 0.00	Celiac disease
26	Control	0.00 : 1.00	Control
27	Control	0.00 : 1.00	Control

Table S2. *Cont.*

**Unsupervised Analysis for Intestinal Biopsies**			
**Subject**	**Diagnosis**	**CD vs. Control Probability**	**Classification**
28	Control	0.00 : 1.00	Control
29	Control	0.00 : 1.00	Control
30	Control	0.00 : 1.00	Control
31	Control	0.00 : 1.00	Control
32	Control	0.33 : 0.67	Control
33	Control	0.00 : 1.00	Control
34	Control	0.00 : 1.00	Control
35	Control	0.33 : 0.67	Control
36	Control	0.00 : 1.00	Control
37	Control	0.00 : 1.00	Control
38	Control	0.00 : 1.00	Control
39	Control	0.00 : 1.00	Control
40	Control	0.00 : 1.00	Control
41	Control	0.00 : 1.00	Control
42	Control	0.00 : 1.00	Control
43	Control	0.00 : 1.00	Control
44	Control	0.00 : 1.00	Control
45	Control	0.33 : 0.67	Control
46	Control	0.00 : 1.00	Control
47	Control	0.33 : 0.67	Control
48	Control	0.00 : 1.00	Control
49	Control	0.50 : 0.50	Celiac disease *

Celiac disease vs. control probability was calculated (second column) in order to compare the obtained classification with the diagnosis. Asterisks highlight misclassified subjects.

Table S3. Clinical and serological data of the CD patients and controls enrolled in the study.

Parameters	Group 1 (CD Patients)	Group 2 (Controls)
No.	25	33
Sex	11 M, 14 F	16 M, 17 F
Age	9.4 ± 4.4	9.4 ± 3.9
Height SDS	0.5 ± 1.6	−0.31 ± 1.48
BMI SDS	−0.88 ± 1.29	−0.80 ± 1.99
Positive serological test for CD (anti-tTG IgA/IgG and/or anti-endomysial IgA)	25/25	0/33
Confirmatory biopsy for CD	25/25	0/33

Height and body mass index (BMI) are indicated as standard deviation scores (SDS).
